# *Raineya orbicola* gen. nov., sp. nov. a slightly thermophilic bacterium of the phylum Bacteroidetes and the description of *Raineyaceae* fam. nov.

**DOI:** 10.1099/ijsem.0.002556

**Published:** 2018-01-10

**Authors:** Luciana Albuquerque, Ana Rita M. Polónia, Cristina Barroso, Hugo J. C. Froufe, Olga Lage, Alexandre Lobo-da-Cunha, Conceição Egas, Milton S. da Costa

**Affiliations:** ^1^​ Center for Neuroscience and Cell Biology, University of Coimbra, 3004-504 Coimbra, Portugal; ^2^​ Next Generation Sequencing Unit, Biocant, BiocantPark, Núcleo 04, Lote 8, 3060-197 Cantanhede, Portugal; ^3^​ Departamento de Biologia, Faculdade de Ciências, Universidade do Porto, Rua do Campo Alegre s/n° 4169-007 Porto, Portugal; ^4^​ CIMAR/CIIMAR – Centro Interdisciplinar de Investigação Marinha e Ambiental – Universidade do Porto, Rua dos Bragas, 289, 4050-123 Porto, Portugal; ^5^​ Laboratório de Biologia Celular, Instituto de Ciências Biomédicas Abel Salazar, ICBAS, Universidade do Porto, Rua de Jorge Viterbo Ferreira, 228, 4050-313 Porto, Portugal

**Keywords:** new taxa, Bacteroidetes, *Raineyaceae* fam. nov., *Raineya* gen. nov., *Raineya orbicola* sp. nov

## Abstract

An isolate, designated SPSPC-11^T^, with an optimum growth temperature of about 50 °C and an optimum pH for growth between 7.5 and 8.0, was recovered from a hot spring in central Portugal. Based on phylogenetic analysis of its 16S rRNA sequence, the new organism is most closely related to the species of the genus *Thermonema* but with a pairwise sequence similarity of <85 %. The isolate was orange-pigmented, formed non-motile long filaments and rod-shaped cells that stain Gram-negative. The organism was strictly aerobic, oxidase-positive and catalase-positive. The major fatty acids were iso-C_15:0,_ iso-C_15 : 0_ 2-OH and iso-C_17 : 0_ 3-OH. The major polar lipids were one aminophospholipid, two aminolipids and three unidentified lipids. Menaquinone 7 was the major respiratory quinone. The DNA G+C content of strain SPSPC-11^T^ was 37.6 mol% (draft genome sequence). The high quality draft genome sequence corroborated many of the phenotypic characteristics of strain SPSPC-11^T^. Based on genotypic, phylogenetic, physiological and biochemical characterization we describe a new species of a novel genus represented by strain SPSPC-11^T^ (=CECT 9012^T^=LMG 29233^T^) for which we propose the name *Raineya orbicola* gen. nov., sp. nov. We also describe the family *Raineyaceae* to accommodate this new genus and species.

The vast majority of the species of the phylum Bacteroidetes have optimum growth temperatures that range from about 25 °C and 45 °C, while slightly thermophilic or thermophilic species are very rare. Some organisms, such as *Pseudozobellia thermophila* [1] and *Lutaonella thermophila* [2], have slightly elevated optimum growth temperatures of around 40–45 °C, while other species, such as *Anaerophaga thermohalophila*, are slightly thermophilic [3], with an optimum growth temperature of around 50 °C. Two other species classified in the phylum Bacteroidetes are thermophilic, namely *Thermonema lapsum* [4] and *Thermonema rossianum* [5] with optimum growth temperatures of about 60 °C and a maximum growth temperature of around 65 °C. Until recently, the two species of the genus *Rhodothermus*, *Rhodothermus marinus* and *Rhodothermus profundi* [6–9], with optimum growth temperatures of over 65 °C and maximum growth temperatures below 80 °C, were included in the phylum Bacteroidetes but are now classified in the novel phylum named ‘Rhodothermaeota’ [10].

We recently isolated one strain of a slightly thermophilic organism with an optimum growth temperature of around 50 °C and a maximum growth temperature of 60 °C. Phylogenetic analysis of the 16S rRNA gene sequence showed that this organism represents a distinct lineage within the phylum Bacteroidetes. Based on phylogenetic, physiological and biochemical parameters, we are of the opinion that strain SPSPC-11^T^ represents a novel genus and species, for which we propose the name *Raineya orbicola* gen. nov., sp. nov. We also propose that this organism represents a new family for which we propose the name *Raineyaceae* fam. nov.

Strain SPSPC-11^T^ was isolated from a reddish biofilm at the hot spring at São Pedro do Sul in Central Portugal (40° 46′ N, 8° 4′ W). The sample was maintained without temperature control for 1 day, and then 0.001 to 0.1 ml in 10 ml water were filtered through membrane filters (Gelman type GN-6; pore size 0.45 µm; diameter 47 mm). The filters were placed on the surface of solidified *Thermus* medium [11], the plates were wrapped in plastic to prevent evaporation and incubated at 45 °C for up to 5 days. Cultures were purified by sub-culturing and the isolates stored at –70 °C in *Thermus* medium with 15 % (w/v) glycerol.

Unless otherwise stated, all biochemical and tolerance tests were performed, as described previously [12, 13], in liquid *Thermus* medium or on *Thermus* agar plates [11] at 45 °C for up to 7 days, rather than at the optimum growth temperature of about 50 °C, because the cultures remained viable for longer periods of time. Cell morphology and motility were examined by phase contrast microscopy during the exponential growth phase. For transmission electron microscopy (TEM), bacteria were fixed for 2 h with 2.5 % glutaraldehyde in 0.1 M cacodylate buffer (pH 7.2), washed in buffer, postfixed for 4 h with buffered 2 % OsO_4_, washed in buffer, followed by 1 h in 1 % uranyl acetate, dehydrated in ethanol and embedded in Epon. Ultrathin sections were stained with uranyl acetate and lead citrate. For scanning electron microscopy (SEM), bacteria were initially processed as for TEM, but after postfixation a drop of bacteria suspended in buffer was laid on each coverslip coated with poly-lysine. After resting for 15 min with the buffer, the bacteria on the coverslips were dehydrated in ethanol and critical-point dried. Samples were coated with Au before being observed.

The presence of flexirubin-type pigments was determined by flooding bacterial cells with 20 % KOH [14]. The absorption spectra of pigments extracted using acetone/methanol 7 : 2 (v/v) were determined at 200–900 nm with a UV–visible spectrophotometer (ThermoScientific). The growth temperature range of the strain was examined at 5 °C increments between 30 and 65 °C by measuring the turbidity (610 nm) of cultures incubated in 300 ml metal-capped Erlenmeyer flasks, containing 100 ml medium in a rotary water-bath shaker at 150 r.p.m. The pH range for growth was examined at 45 °C in the same medium by using 50 mM MES, HEPES, TAPS and CAPSO over a pH range of 6.0 to 9.0 with 0.5 unit increments, in a rotary water-bath shaker. Growth with added salt, 1 % (w/v) NaCl, was determined in liquid medium. Catalase, oxidase and DNAse activities were examined as described previously [12, 13]. Additional characteristics were obtained using the API ZYM system (bioMérieux) at 45 °C. Anaerobic growth was assessed in culture medium containing KNO_3_ (1.0 g l^−1^) incubated in anaerobic chambers (GENbox anaer, bioMérieux). Results were recorded after 30 days of incubation at 45 °C. Single-carbon source assimilation tests were performed in a minimal medium composed of *Thermus* basal salts containing filter-sterilized single carbon sources (2.0 g l^−1^), ammonium sulfate (0.5 g l^−1^) and a vitamin and nucleotide solution at a final concentration of 40 µg l^−1^ [15] consisting of thiamine, riboflavin, pyridoxine, biotin, folic acid, inositol, nicotinic acid, pantothenic acid, p-aminobenzoic acid, cyanocobalamin, adenine, thymine, cytosine, guanine, cytidine, uracil and inosine (10 ml l^−1^). Growth of the strain on single carbon sources was examined by measuring the turbidity of cultures in 20 ml screw capped tubes containing 10 ml medium for up to 7 days.

The polar lipids were extracted from freeze-dried cells and the individual polar lipids were separated by two-dimensional thin-layer chromatography. To visualize phospholipids, aminolipids, glycolipids and total lipids, the following reagents were used, respectively, molybdenum blue, ninhydrin, *α*-naphthol-sulfuric acid and molybdophosphoric acid [16]. Lipoquinones were extracted from freeze-dried cells and purified by thin-layer chromatography. The purified lipoquinones were separated by high-performance liquid chromatography (HPLC) as described previously [17]. Cultures for fatty acid analysis were grown in *Thermus* liquid medium at 45 °C for 5, 8 and 24 h. Fatty acid methyl esters were obtained from fresh wet biomass, separated, identified and quantified with the standard MIS Library Generation Software, version 6.0, aerobe TSBA method (Microbial ID Inc., MIDI) as described previously [18].

Total genomic DNA was extracted following the method of Nielsen *et al.* [19], and used for the different analyses performed. The G+C content of DNA was determined by HPLC as described by Mesbah *et al.* [20] and by genome sequencing (see below). PCR-amplification of 16S rRNA genes was carried out as described by Rainey *et al.* [21]. The 16S rRNA gene sequence was determined by Sanger sequencing (Macrogen).

The genomic DNA was prepared with the Nextera XT DNA Library Preparation Kit and sequenced using paired-end 2×300 bp on the MiSeq system (Illumina). Sequenced reads were quality filtered with Trimmomatic [22] and assembled with SPAdes (version 3.7.1; [23]) and the resulting contigs annotated with prokaryotic genome prediction [24]. Genome estimated completeness and contamination were verified with CheckM (version 1.0.7) [25]. RNAmmer (version 1.2) [26] and Usearch61 [27] (against Greengenes database, version 13.8) were used for complete or partial 16S rRNA genes analysis. The two 16S rRNA genes identified were scattered in three contigs, but the complete ribosomal genes were manually reconstructed based on the mapping of paired-end reads against the assembled contigs by using Bowtie 2 [28] The genome of strain SPSPC-11^T^ was compared to the genomes of several organisms of the order Cytophagales, namely *Bacteroides fragilis* YCH46 (NC_006347.1), *Hymenobacter roseosalivarius* DSM 11622^T^ (GCA_900176135.1), *Cyclobacterium marinum* DSM 745^T^ (NC_015914), *Cytophaga hutchinsonii* ATCC 33406^T^ (NC_008255.1) and *Thermonema rossianum* DSM 10300^T^ (NZ_AUGC00000000) with GET_HOMOLOGUES using blastp and OrthoMCL [29]. Orthologous genes were annotated against the Kyoto Encyclopedia of Genes and Genomes and assigned to metabolic pathways (sequence similarity cutoff e-values of 1e^−5^) using kobas 2.0 [30].

Isolate SPSPC-11^T^ formed Gram-negative non-motile short rod-shaped cells and long filaments during the exponential phase of growth (Fig. 1a, b). Cell-wall septa were rarely seen to divide into smaller cells (Fig. 1c). The bacterium had a Gram-negative type of cell wall (Fig. 1d) and a few small electron-dense inclusions could be seen in the cytoplasm. Colonies were orange-pigmented on *Thermus* medium.

**Fig. 1.**
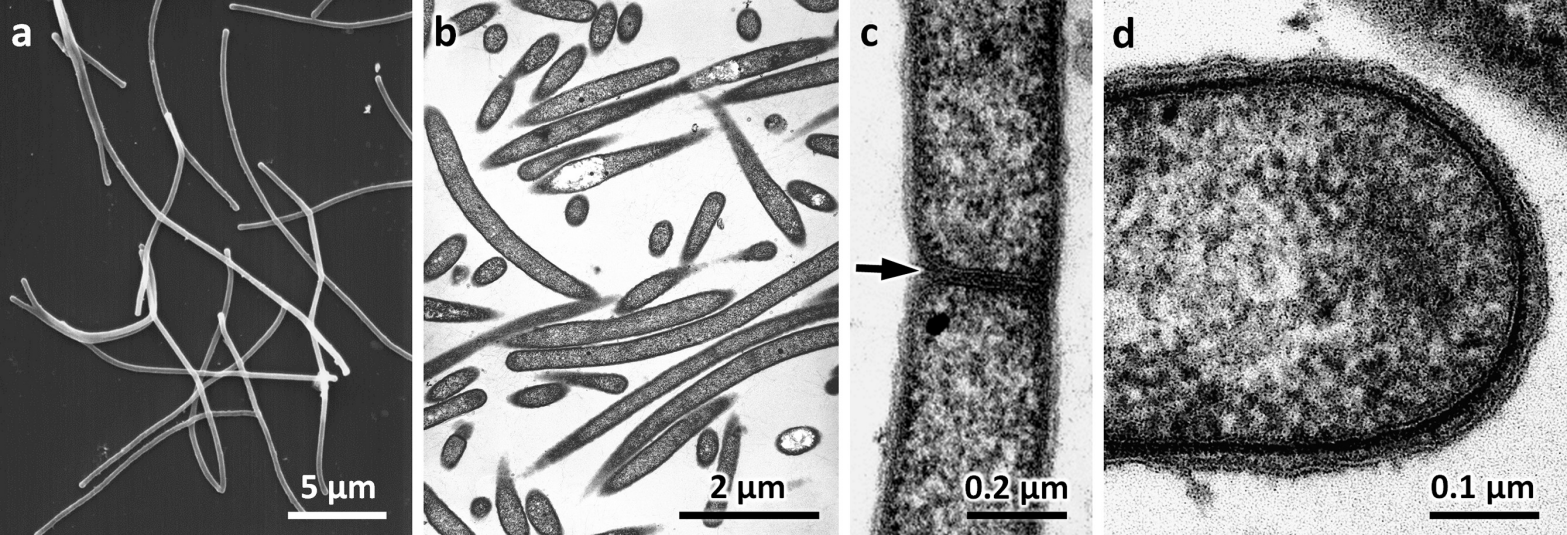
Electron microscopy by SEM and TEM of exponential phase cells of strain SPSPC-11^T^. (a) Filamentous cells from a young culture (2–5 h) observed by SEM. (b) Filamentous cells from a young culture (2–5 h) observed by TEM. (c) A septum is indicated by an arrow. (d) High magnification showing the Gram-negative type of cell wall.

Strain SPSPC-11^T^ had an optimum growth temperature of about 50 °C; growth occurred at 35 and 60 °C. The optimum pH for growth was about 7.5–8.0 with a range of growth between pH 6.5 and 8.5. The isolate did not utilize any of the sugars tested and only a few amino acids, but grew well on casamino acids, tryptone, peptone and yeast extract (Table 1). Yeast extract or a vitamin and nucleotide supplements were necessary for growth in minimal medium. The polar lipid pattern on thin-layer chromatography of the new organism revealed the presence of aminolipids, aminophospholipids and unidentified lipids (Fig. S1, available in the online version of this article). The major respiratory lipoquinone was menaquinone 7. The major fatty acids of these organisms were iso-C_15 : 0_, iso-C_15 : 0_ 2-OH and iso-C_17 : 0_ 3-OH, and were similar during several phases of growth despite the notable changes in morphology (Table S1).

**Table 1.** Distinguishing characteristics between strain SPSPC-11^T^, *Thermonema lapsum* DSM 5718^T^ and *Thermonema rossianum* DSM 10300^T^ Strains: 1, SPSPC-11^T^; 2, *Thermonema lapsum* DSM 5718^T^, 3, *Thermonema rossianum* DSM 10300^T^. All strains were catalase- and oxidase-positive. Strain SPSPC-11^T^ and *Thermonema rossianum* DSM 10300^T^ do not reduce nitrate. In the API ZYM test strips strain SPSPC-11^T^ is positive for alkaline phosphatase, esterase (C4), esterase lipase (C8), lipase (C14), leucine arylamidase, valine arylamidase, cystine arylamidase, trypsin, *α*-chymotrypsin, acid phosphatase and naphthol-AS-BI-phosphohydrolase, but negative for *α*-galactosidase, *β*-galactosidase, *β*-glucuronidase, *α*-glucosidase, *β*-glucosidase*, N*-acetyl-*β*-glucosaminidase, *α*-mannosidase and *α*-fucosidase. Strain SPSPC-11^T^ does not hydrolyse DNA, aesculin and arbutin. All strains hydrolyse casein, gelatin and hippurate but none of the strains hydrolyse starch and xylan. All strains assimilate casamino acids and yeast extract but do not assimilate d-glucose, d-fructose, d-galactose, d-mannose, l-rhamnose, l-fucose, l-sorbose, d-ribose, d-xylose, d-arabinose, l-arabinose, sucrose, maltose, cellobiose, lactose, trehalose, raffinose, melibiose, methyl *α*-d-glucopyranoside, glycerol, ribitol, xylitol, sorbitol, d-mannitol, *myo*-inositol, erythritol, d-arabitol, *α*-ketoglutarate, dl-lactate, succinate, malate, citrate, benzoate, fumarate, formate, d-gluconate, d-glucoronate, l-asparagine, glycine, l-histidine, l-lysine, l-arginine, l-valine, l-phenylalanine, l-leucine, l-isoleucine, l-ornithine, l-methionine, l-threonine, l-glucosamine, *N*-acetylglucosamine, cysteine, cystine, tyrosine, tryptophan, glycine-betaine and dextrin. +, Positive; –, negative; nd, not determined.

**Characteristic**	**1**	2*†	**3**†
Cell size (µm)	0.5–0.8×5.0–15.0	0.25–0.3×60	0.7 wide
Temperature for growth (°C)			
Optimum	50	60	60
Range	35–60	35–65	35–65
pH for growth			
Optimum	7.5–8.0	6.5	7.0–7.5
Range	6.5–8.5	nd	5.5–9.5
NaCl for growth (%)			
Optimum	0	0	1–3
Range	0	0–3	0.5–5
Assimilation of:			
Acetate	+	–	–
Pyruvate	+	–	–
Aspartate	+	–	–
l-Glutamate	+	–	–
l-Alanine	+	–	–
l-Proline	+	–	–
l-Glutamine	+	–	–
l-Serine	+	–	–
Tryptone	+	–	–
Peptone	+	+	nd
G+C content (mol%) (HPLC)	39.2	47.0	50.9

*Data from Hudson *et al.* [4].

†Data from Tenreiro *et al*. [5].

The analysis of the 16S rRNA gene sequence of strain SPSPC-11^T^ (KY990922) using the EzBioCloud database version 2017.5 [31] demonstrated that strain SPSPC-11^T^ belonged to the phylum Bacteroidetes and represented a novel cultured lineage that shared less than 85 % similarity with previously described taxa. The SPSPC-11^T^ lineage clusters with the lineage of the family *Thermonemataceae* within the order Cytophagales (Fig. 2). Comparison of the two 16S rRNA gene sequences (MF125287, M125288) determined from the draft genome sequence with environmental sequences showed it to share 90–99 % similarity with sequences recovered from a range of aquatic environments (Fig. 3 and Table S2).

**Fig. 2.**
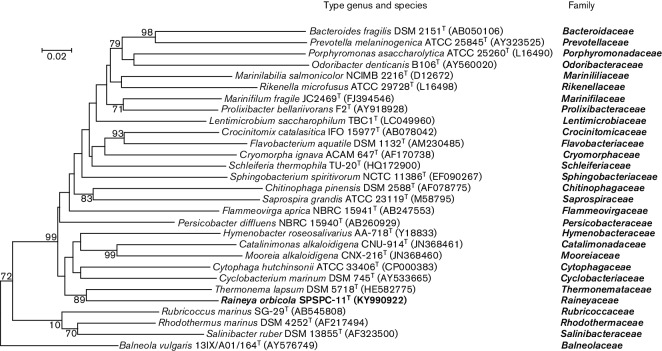
Phylogenetic position of strain SPSPC-11^T^ within the radiation of representatives of the families of the phyla Bacteroidetes and ‘Rhodothermaeota’. The phylogenetic dendrogram was generated using the neighbour-joining method [38] in mega 6.0 [39]. Bootstrap values, expressed as percentages of 1000 replications, are given at branching points. Bar, 2 inferred nucleotide substitutions per 100 nucleotides.

Recently, published studies on the phylogeny of the phylum Bacteroidetes, based on whole genome comparisons, have demonstrated the existence of a number of lineages representing new taxa at the phylum, class, order and family levels [10, 32], although Munoz *et al*. [10] designated 16S rRNA gene sequence similarity ranges outside the taxonomic levels proposed by Hahnke *et al*. [32]. Phylogenetic analysis of the 16S rRNA gene sequence of strain SPSPC-11^T^ showed its position within this classification of the phylum Bacteroidetes and related taxa (Fig. 2). Based on the 16S rRNA gene sequence similarity values to related taxa (<85 %) and the position within the phylogenetic tree it is demonstrated that strain SPSPC-11^T^ represents a novel lineage at the family level within the order Cytophagales.

The observation that strain SPSPC-11^T^ was unable to grow on any of the sugars examined prompted us to produce a high-quality draft genome sequence to assess the possibility that some genes involved in sugar catabolism would not be present. Additionally, the genome was searched for other metabolic processes and compared with the genomes sequences of carbohydrate-utilising Cytophagales species that assimilate carbohydrates, namely *Bacteroides fragilis* YCH46 (NC_006347.1), *Hymenobacter roseosalivarius* DSM 11622^T^ (GCA_900176135.1), *Cyclobacterium marinum* DSM 745^T^ (NC_015914) and *Cytophaga hutchinsonii* ATCC 33406^T^ (NC_008255.1), as well as the genome sequence of *Thermonema rossianum* DSM 10300^T^ (NZ_AUGC00000000) that does not utilize any sugars tested [5].

The SPSPC-11^T^ DNA sequence run generated 2 112 714 paired-end reads of which 1 796 859 high quality reads remained after quality filtering. The *de novo* read assembly produced 104 contigs with an N50 size of 67 061 bp (Table 2). The high-quality draft assembled genome sequence consisted of 3 070 213 bp with a DNA G+C content of 37.6 mol%. CheckM estimated the genome to be near-completion (98.2 %) and the level of contamination to be extremely low (0.3 %). No contamination was detected for 16S rRNA genes as tested by RNAmmer and Usearch61. The genome had a total of 2730 genes, including 2685 protein-coding genes, 39 tRNA genes and 6 rRNA genes (two 5S, two 16S and two 23S) (Table 2). Analysis of the whole-genome sequence demonstrated the presence of two 16S RNA gene-coding sequences. The two 16S rRNA gene sequences differed at eight positions over 1501 compared nucleotides representing 99.47 % identity. The presence of multiple 16S rRNA gene copies with such levels of similarity between the gene copies of the same organism have been reported across many bacterial taxa and in representatives of the phylum Bacteroidetes [33, 34].

**Table 2.** Genome sequencing project information and statistics Strains: 1, SPSPC-11^T^; 2, *Thermonema rossianum* DSM 10300^T^.

MIGS ID†	Attribute	Value/comment	
		**1**	2*
MIGS-28	Libraries used	Illumina paired-end library (2×300 bp insert size)	Illumina paired-end library
MIGS 29	Sequencing platforms	Illumina MiSeq	Illumina HiSeq 2000 and HiSeq2500
	Size of raw data included in the assembly process (Mbp)	820	176.2
MIGS 30	Assembler	Spades version 3.7.1	‒
MIGS 31	Finishing quality	High-quality draft	High-quality draft
MIGS 31.2	Sequencing depth of coverage	250×	‒
MIGS 31.3	Number of contigs	104	26
MIGS 32	Gene calling method	PGP	Prodigal 2.5
	N50 (bp)	67 061	202 966
	Estimated genome completeness (%)	98.2	‒
	Assembled genome size (bp)	3 070 213	2 956 866
	DNA coding (bp)	2 806 590	2 723 503
	DNA G+C (bp)	1 151 283	1 441 896
	DNA G+C (mol%)	37.6	48.6
	Total genes	2730	2654
	Protein-coding genes	2685	2599
	RNA genes	45	55
	tRNA genes	39	44
	rRNA genes	6	9
	5S	2	3
	16S	2	3
	23S	2	3
	Genes with function prediction	2115	1935
	Genes assigned to COGs	1320	1511
	Genes with Pfam domains	2048	1998
	Genes with Tfam domains	749	‒
	CRISPR repeats	4	2
	Estimated contamination (%)	0.3	‒
	Authenticity of strain checked by	16S (rRNA gene from Sanger and genome sequencing)	‒
	Accession number of the assembly	NKXO00000000	ASM42682v1
	Accession number of raw data the assembly	SRR5815076	SRP054817

*Data from NCBI Bioproject PRJNA195851 and JGI Project 1015836.

†Based on MIGS recommendations [40].

The draft genome comprised 2115 genes with putative functions (~79 % of total protein-coding genes) and 1320 allocated to COG functional categories (~49 % of total protein-coding genes). The most abundant COG category was ‘Translation, ribosomal structure, and biogenesis’ followed by ‘Cell wall/membrane biogenesis’ and ‘Amino acid transport and metabolism’ (Table S3).

Several genes coding for enzymes involved in the initial catabolism of carbohydrates to glucose were not identified in the new strain, thus preventing the utilization of hexoses or pentoses through the Embden–Meyerhof–Parnas or the Entner–Doudoroff pathways. It is noteworthy that *T. rossianum*, also lacks the same genes for the initial catabolism of sugars and is, like strain SPSPC-11^T^, unable to grow on any of the sugars examined [5]. In contrast, the genome sequence of *B. fragilis*, *H. roseosalivarius*, *Clb. marinum* and *Cyt. hutchinsonii* predict the assimilation of hexoses and pentoses through these pathways, as also confirmed by assimilation tests [35–37].

It is possible that strain SPSPC-11^T^ lacks the genetic ability to metabolize carbohydrates, confirming the results of the phenotypic tests that show that sugars do not serve as carbon and energy sources for growth. Similar to other members of the order Cytophagales (*B. fragilis*, *H. roseosalivarius*, *Clb. marinum* and *Cyt. hutchinsonii*), the putative gene for fructose-1,6-bisphosphatase (EC:3.1.3.11) was identified, suggesting that strain SPSPC-11^T^ can perform gluconeogenesis. The genome sequence of strain SPSPC-11^T^ predicts that the tricarboxylic acid cycle is complete.

The draft genome of strain SPSPC-11^T^ indicated that oxidative phosphorylation occurs via NADH dehydrogenase, succinate dehydrogenase, cytochrome c, cytochrome c oxidase and an F-type ATPase. The *T. rossianum* genome sequence appears to possess several genes coding for the same oxidative phosphorylation functions that were identified in the strain SPSPC-11^T^ with the exception of the NuoEG subunits of the NADH dehydrogenase complex. In contrast to strain SPSPC-11^T^, genes coding for cytochrome bd complex were identified in *Clb. marinum* and *B. fragilis.* The genome of *B. fragilis* lacks not only cytochrome c oxidase-like genes but also the NuoEFG subunits of the NADH dehydrogenase complex. The latter organisms also possess some V/A Type ATPase-associated genes in addition to F-type ATPase.

The absence of assimilatory nitrate or dissimilatory nitrite reduction genes by strain SPSPC-11^T^, *H. roseosalivarius* and *T. rossianum* confirms the absence of phenotypic nitrate reduction. The genes involved in nitrate/nitrite transport and nitrate reduction, namely the assimilatory nitrate reductase and the enzymes for denitrification, were not encountered. The other Cytophagales, namely *B. fragilis, Clb. marinum* and *Cyt. hutchinsonii*, possess putative genes involved in nitrite reduction, while *Clb. marinum* and *Cyt. hutchinsonii* also had genes involved in the assimilatory nitrate reduction to nitrite.

From the comparison of environmental sequences from uncultured organisms it was demonstrated that strain SPSPC-11^T^ is a cultured representative of a family level phylogenetic lineage within the phylum Bacteroidetes that has been already detected and is represented by 16S rRNA gene sequences recovered from geographically distant aquatic environments, many of them geothermal (Fig. 3 and Table S2). Based on the 16S rRNA gene sequence similarities within the lineage represented by environmental sequences and now strain SPSPC-11^T^ it is clear that this lineage contains a number of novel genera and species yet to be cultured. Phylogenetic analysis demonstrated that strain SPSPC-11^T^ represents the first cultured member of a novel family level lineage within the order *Cytophagales* of the phylum Bacteroidetes (Figs 2 and 3).

**Fig. 3.**
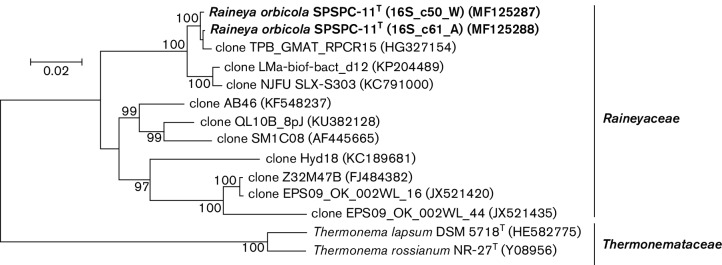
Phylogenetic position of strain SPSPC-11^T^ within the radiation of representatives of environmental clone sequences to belong to the *Raineya* lineage. The source of the environmental clone sequences is shown in Table S2. The phylogenetic dendrogram was generated using the neighbour-joinging method [38] in mega 6.0 [39]. Bootstrap values, expressed as percentages of 1000 replications, are given at branching points. Bar, 2 inferred nucleotide substitutions per 100 nucleotides.

The new lineage represented by strain SPSPC-11^T^ possesses genotypic and phenotypic features that resembled those of the species of *Thermonema*. However, notable differences include amino acid assimilations: strain SPSPC-11^T^ assimilates some single amino acids while the *Thermonema* species assimilate only complex mixtures of amino acids; the optimum growth temperatures of the organisms differ by about 10 °C; the inability of new species to grow in medium with added NaCl and the large difference between the DNA G+C mol% of strain SPSPC-11^T^ and the species of *Thermonema* (Table 1).

On the basis of these results, we propose that strain SPSPC-11^T^ represents a novel species of a new genus for which we recommend the name *Raineya orbicola* gen. nov., sp. nov. Moreover, we are of the opinion that the genotypic, phylogenetic, chemotaxonomic and phenotypic characteristics warrant a new family within the phylum Bacteroidetes for which we propose the name *Raineyaceae* fam. nov.

## Description of *Raineya* gen. nov.

*Raineya* (Rai.ney.a. N.L. fem. n. *Raineya* referring to Fred A. Rainey, for his contributions to the taxonomy and phylogeny of archaea and bacteria).

Oxidase- and catalase-positive. Flexirubin-type pigments are not present. Carbohydrates are not utilized for growth. The polar lipid profile is composed of aminolipids, aminophospholipids and unidentified lipids. The fatty acid composition is dominated by iso-branched fatty acids and hydroxyl fatty acids. The type species of the genus is *Raineya orbicola*.

## Description of *Raineya orbicola* sp. nov.

*Raineya orbicola* (or.bi′co.la. L. n. *orbis*, the whole world; L. suff. *cola*, inhabitant, dweller; N.L. n. *orbicola*, inhabitant of the whole world).

Forms long filaments and rod-shaped cells 0.5–0.8 µm wide and 5.0–15.0 µm long; colonies on *Thermus* medium are orange-pigmented due to carotenoids. Growth occurs between 35 and 60 °C; the optimum growth temperature is about 50 °C. The optimum pH for growth is about 7.5–8.0; growth occurs between pH 6.5 and 8.5. Optimum growth occurs without added NaCl; no growth occurs with 1 % NaCl. Yeast extract or a vitamin and nucleotide solution is required for growth. Nitrate is not reduced to nitrite. Gelatine, casein and hippurate are degraded; starch, aesculin, arbutin and xylan are not degraded. DNAse negative. In the API ZYM alkaline phosphatase, esterase (C4), esterase lipase (C8), lipase (C14), leucine arylamidase, valine arylamidase, cystine arylamidase, trypsin, *α*-chymotrypsin, acid phosphatase and naphthol-AS-BI-phosphohydrolase are positive; other enzyme activities are negative. Acetate, pyruvate, aspartate, l-glutamate, l-alanine, l-proline, l-glutamine, l-serine, yeast extract, tryptone, peptone and casamino acids are assimilated. Other single carbon sources tested are not assimilated (Table 1). The major fatty acids are iso-C_15 : 0_, iso-C_15 : 0_ 2-OH and iso-C_17 : 0_ 3-OH. The DNA of strain SPSPC-11^T^ has a G+C content of 39.2 mol% (HPLC method) and 37.6 mol% (genome sequencing). The type strain SPSPC-11^T^ (=CECT 9012=LMG 29233) was isolated from a hot spring at São Pedro do Sul in Central Portugal.

## Description of *Raineyaceae* fam. nov.

*Raineyaceae* (Rai.ney.a.ce′ae. N.L. fem. dim. n. *Raineya*, type genus of the family; suff. -*aceae*, ending denoting a family; N.L. fem. pl. *Raineyaceae*, the *Raineya* family).

Cells stain Gram-stain-negative and form rod-shaped cells. Endospores are not formed. Organotrophic and strictly aerobic. Slightly thermophilic. Menaquinone 7 is the major respiratory lipoquinone. Represents a distinct phylogenetic lineage within the order Cytophagales. The type genus of this family is *Raineya*.

## Supplementary Data

232 Supplementary File 1
