# Supplementary material for: Raineya orbicola gen. nov., sp. nov. a slightly thermophilic bacterium of the phylum Bacteroidetes and the description of Raineyaceae fam. nov
Source: Int J Syst Evol Microbiol. 2018 Jan 10;68(4):982–9. doi: 10.1099/ijsem.0.002556 (PMC5982127; doi:10.1099/ijsem.0.002556)
Supplement: Supplementary File 1 [file ijsem-68-982-s001.pdf]

Raineya orbicola gen. nov., sp. nov. a slightly thermophilic bacterium of the phylum Bacteroidetes and the description of *Raineyaceae* fam. nov.

Luciana Albuquerque[1], Ana Rita M. Polónia[1], Cristina Barroso[1,2], Hugo J. C. Froufe[2], Olga Lage[3,4], Alexandre Lobo-da-Cunha[4,5], Conceição Egas[1,2] and Milton S. da Costa[1*]

[1]Center for Neuroscience and Cell Biology, University of Coimbra, 3004-504 Coimbra, Portugal

[2]Next Generation Sequencing Unit, Biocant, BiocantPark, Núcleo 04, Lote 8, 3060-197 Cantanhede, Portugal

[3]Departamento de Biologia, Faculdade de Ciências, Universidade do Porto, Rua do Campo Alegre s/nº 4169-007 Porto, Portugal

[4]CIMAR/CIIMAR – Centro Interdisciplinar de Investigação Marinha e Ambiental – Universidade do Porto, Rua dos Bragas, 289, 4050-123 Porto, Portugal

[5]Laboratório de Biologia Celular, Instituto de Ciências Biomédicas Abel Salazar, ICBAS, Universidade do Porto, Rua de Jorge Viterbo Ferreira, 228, 4050-313 Porto, Portugal

*Corresponding author

Milton S. da Costa

Tel: +351-239829802 / Fax: +351-239822776

E-mail: milton@ci.uc.pt

Short running title: *Raineya* gen. nov., sp. nov.

Figure Legend:

Fig. S1. Two-dimensional thin-layer chromatography of polar lipids of strain SPSPC-11^T grown at 45 ºC. The lipids were stained by spraying with 5 % molybdophosphoric acid in ethanol followed by heating at 160 ºC. AL1,2,3, aminolipids 1,2,3; APL1,2, aminophospholipids 1,2; UL1,2,3,4,5, unidentified lipids 1,2,3,4,5.

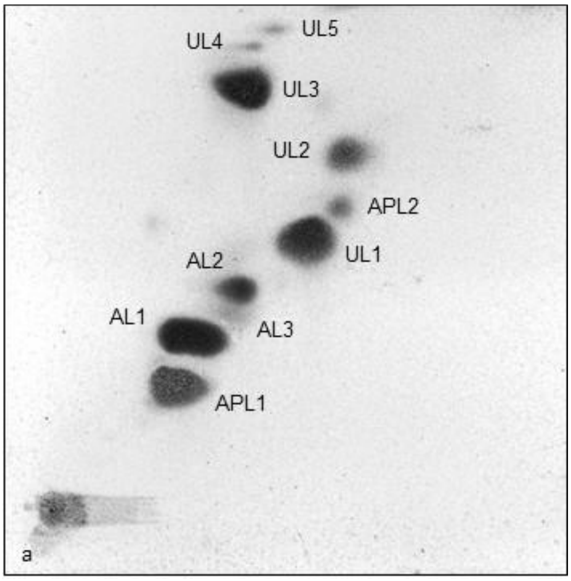

Fig. S1.

Table S1. Fatty acid composition of strain SPSPC-11^T grown in *Thermus* liquid medium at 45 ºC, and *Thermonema lapsum* DSM 5718^T and *Thermonema rossianum* DSM 10300^T grown on Degryse medium 162 [5] agar plates at 60 ºC.

Strains: 1, SPSPC-11^T; 2, *Thermonema lapsum* DSM 5718^T, 3, *Thermonema rossianum* DSM 10300^T. Results are percentage of the total fatty acids (mean ± SD of two to four analyses); values for fatty acids present at levels of less than 0.5 % are not shown or are represented by tr (trace amount); ECL, equivalent chain length.

			1		2*	3*
**Fatty acids**	**ECL**	**5 h**	**8 h**	**24 h**	**24 h**	**24 h**
Unknown 11.543	11.543	0.7 ± 0.1	0.6 ± 0.1	0.6 ± 0.1	–	–
iso-$C_{14:0}$	13.619	–	–	–	1.0	1.5
Unknown 13.565	13.565	5.6 ± 0.4	4.9 ± 0.5	4.5 ± 0.7	–	–
iso-$C_{15:0}$	14.623	59.4 ± 1.5	61.8 ± 1.6	57.5 ± 1.7	37.4	39.9
anteiso-$C_{15:0}$	14.713	1.2 ± 0.2	1.1 ± 0.2	1.4 ± 0.2	8.4	8.6
$C_{15:0}$	15.000	1.0 ± 0.2	1.0 ± 0.2	1.4 ± 0.2	2.7	1.8
iso-$C_{16:0}$	15.627	tr	tr	tr	1.0	1.5
iso-$C_{15:0}$ 2-OH	15.852	8.7 ± 0.4	8.1 ± 0.3	10.8 ± 0.6	7.4	5.4
$C_{16:0}$	16.000	3.7 ± 0.3	3.6 ± 0.2	2.9 ± 0.3	1.2	1.2
iso-$C_{15:0}$ 3-OH	16.134	2.8 ± 0.2	2.9 ± 0.2	3.0 ± 0.2	8.9	8.3
$C_{15:0}$ 2-OH	16.219	tr	tr	tr	1.2	0.6

iso-$C_{17:0}$	16.630	tr	tr	tr	1.1	1.2
Unknown 16.582	16.582	0.7 ± 0.1	0.6 ± 0.1	0.6 ± 0.1	2.0	2.1
$C_{17:1}$ $\omega 6c$	16.860	–	–	–	1.3	0.8
iso-$C_{16:0}$ 3-OH	17.150	tr	tr	tr	1.1	1.4
$C_{16:0}$ 2-OH	17.233	0.5 ± 0.1	0.3 ± 0.1	0.5 ± 0.1	–	–
$C_{16:0}$ 3-OH	17.519	1.9 ± 0.3	1.9 ± 0.3	2.4 ± 0.4	1.5	1.3
iso-$C_{17:0}$ 2-OH	17.880	–	–	–	1.1	0.5
iso-$C_{17:0}$ 3-OH	18.161	10.1 ± 0.7	10.0 ± 0.6	10.1 ± 0.9	17.4	18.7
anteiso-$C_{17:0}$ 3-OH	18.254	–	–	–	0.7	1.0

*Data from Tenreiro *et al*. [5].

Table S2. Information on environmental clone sequences that belong to the *Raineyaceae* lineage within the Bacteroidetes.

Sequence accession	Clone designation	% Identity to SPSPC-11^T	Query cover	Source and location
HG327154	TPB_GMAT_RPCR15	99%	93%	Microbial mat, Tapoban hot springs, Garhwal, India
KP204489	LMa-biof-bact_d12	98%	99%	Microbial biofilm, acidic geothermal area of Copahue, Neuquen, Argentina
KC791000	NJFU SLX-S303	98%	95%	Whitewater of paper making machine, ChangShu, China
KF548237	AB46	93%	98%	Anerobic tank of wastewater treatment plant, China
KU382128	QL10B_8pJ	93%	98%	Microbial mat, Queen's Laundry hot spring, Yellowstone National Park, USA
AF445665	SM1C08	92%	99%	Travertine deposition, Angel Terrace, Mammoth Hot Springs, USA
FJ484382	Z32M47B	92%	91%	Wall biomat, phreatic sinkhole, El Zacaton, Mexico
JX521420	EPS09_OK_002WL_16	92%	99%	Terrestrial sulfidic spring, Sulphur Springs, Oklahoma, USA
KC189681	Hyd18	90%	100%	Freshwater spring, Wakulla Spring, Florida, USA
JX521435	EPS09_OK_002WL_44	89%	99%	Terrestrial sulfidic spring, Sulphur Springs, Oklahoma, USA
*HG327161	TPB_GMAT_RPCR32	100%	55%	Microbial mat, Tapoban hot springs, Garhwal, India
*EU815166	yang-W129	98%	58%	Thermal vent boiling pool, Tibet, China

*not included in phylogenetic analysis and Figure 3 due to short sequences and low query coverage.

Table S3. Number of genes associated with general COG functional categories.

Code	Value	%*	Description
J	135	5.03	Translation, ribosomal structure and biogenesis
K	56	2.09	Transcription
L	95	3.54	Replication, recombination and repair
B	1	0.04	Chromatin structure and dynamics
D	19	0.71	Cell cycle control, Cell division, chromosome partitioning
V	25	0.93	Defence mechanisms
T	45	1.68	Signal transduction mechanisms
M	116	4.32	Cell wall/membrane biogenesis
N	3	0.11	Cell motility
U	21	0.78	Intracellular trafficking and secretion
O	61	2.27	Posttranslational modification, protein turnover, chaperones
C	82	3.05	Energy production and conversion
G	41	1.53	Carbohydrate transport and metabolism
E	112	4.17	Amino acid transport and metabolism
F	53	1.97	Nucleotide transport and metabolism
H	77	2.87	Coenzyme transport and metabolism
I	71	2.64	Lipid transport and metabolism
P	54	2.01	Inorganic ion transport and metabolism
Q	30	1.12	Secondary metabolites biosynthesis, transport and catabolism
R	139	5.18	General function prediction only
S	84	3.13	Function unknown
-	1365	50.84	Not in COGs

* The percentage is based on the total number of protein-coding genes in the annotated genome.
